# Using diffusion of innovation theory to describe perceptions of a passive positioning alarm among persons with mild dementia: a repeated interview study

**DOI:** 10.1186/s12877-016-0183-8

**Published:** 2016-01-08

**Authors:** Annakarin Olsson, Kirsti Skovdahl, Maria Engström

**Affiliations:** Faculty of Health and Occupational Studies, University of Gävle, 80176 Gävle, Sweden; Faculty of Health Sciences, Buskerud and Vestfold University College, P.O. Box 235, 3603 Kongsberg, Norway; Department of Public Health and Caring Sciences, Uppsala University, Box 564, 751 22 Uppsala, Sweden

**Keywords:** Dementia, GPS, Outdoors, Promoting health, Qualitative research, Tracking technology

## Abstract

**Background:**

Problems with memory and decline in cognitive abilities are common during development of dementia. Different kinds of technologies may be useful in supporting persons with dementia and their relatives in daily life. Tracking technologies have the potential to improve independence among persons with dementia. Consequently, the aim of the present study was to describe perceptions of a passive positioning alarm (PPA) among persons with mild dementia.

**Methods:**

A repeated interview study was conducted in Sweden with a strategic sample of 11 persons with mild dementia. Roger’s Diffusion of Innovation Theory was used to deductively analyse the data.

**Results:**

Regarding the *advantages* of the PPA, participants described perceived safety and security for, both themselves, and their relatives, as well as freedom and independence. However, they also expressed concern about the cost of the PPA, reflected on who might be the receiver of the alarm from the PPA, emphasized the importance of opportunities to test the device before becoming a user and early introduction before their problems start, thus allowing them to decide for themselves.

**Conclusions:**

Supporting persons with dementia in their own homes using, e.g., a PPA may enable them and their relatives to remain longer in their own homes and be safer in their own neighbourhoods.

## Background

Problems with memory and decline in cognitive abilities are common during development of a dementia [1]. Different kinds of technologies may be useful in supporting persons with dementia (PwDs) and their relatives in daily life [2]. Technologies used in the care of older persons and PwDs are diverse and can be used from different perspectives, for instance, that of the older persons/PwDs themselves, their relatives and/or healthcare staff; they can serve several purposes as well, for example, to facilitate independent living [3–5], safety and security [6–8] and/or well-being and psychological health [9]. Regarding persons with mild dementia (PwMDs), they still have an intellectual autonomy to decide their interest of using different kinds of technology that are or could be useful for them in the future. They can properly describe their perception of different kinds of technologies and decide for themselves whether or not they would like to use the technology now and/or in the future. Earlier studies have described the importance of early introduction of technology [10, 11] as well as relatives’ desire to know the PwDs’ will regarding the use of different kind of technology e.g., tracking systems, in the care of them. However, PwMDs differs from the one that explain the emergence of tracking system in dementia care, that is to say persons who wander, who deny their symptoms, and at high risk of getting lost. Assistive Technology (AT) was defined in the US Assistive Technology Act of 1998 as “technology used by individuals with disabilities in order to perform functions that might otherwise be difficult or impossible” [12]. AT is often promoted as a means of retaining autonomy and quality of life among older people [13], including PwMDs [14], as AT may help them continue to live independently and safely in their own homes [3–6, 15–17]. Tracking technology is one example of an AT, also subsumed under the heading ‘surveillance technology’ [3, 6, 18, 19]. Tracking technology, e.g., a passive positioning alarm (PPA), is based on a global positioning system (GPS) and is able to show the exact position of the tracking device (and the person wearing the device) on a digital map. The tracking technology used here involves a hidden zone, that is, the person is not visible until he/she leaves a predefined area. The PPA sends its position and an alarm to a mobile phone (thus, not to a stationary receiver), which allows the receiving party to see the alarm signal regardless of where he/she is physically located (thus, not just while at home). Most previous studies have looked at relatives’ (e.g., [8, 19]) and/or professionals’ (e.g., [7, 20–23]) and cognitively intact older people’s (e.g., [22, 23]) views on tracking technologies. Studies involving PwMDs’ [6, 16, 24–27] experiences of using tracking technologies have shown that they expressed both hesitance regarding the use of unfamiliar technology, as well as enhanced feelings of safety and less fear and anxiety. Furthermore, design aspects, e.g., the shape of the device, were mentioned [28].

In the present work Roger’s Diffusion of Innovation Theory (DOI) [29] were used to organize the results. The theory, have shown to be useful in studying individuals’ adoption of e.g., new healthcare information technologies [30]. The DOI theory describes different aspects of adoption of an innovation: *Relative Advantage* - the degree to which the user perceives benefits or improvements upon the existing technology by adopting an innovation; *Compatibility* - How consistent the innovation is with the values, experiences, and needs of the potential adopters; *Complexity* - How difficult the innovation is to understand and/or use; *Trialability* - The extent to which the innovation can be tested or experimented with before a commitment to adopt is made; *Observability* - The extent to which the innovation provides tangible results. In the present study, DOI is used to analyse the results and the aim of the study was to describe PwMDs’ perceptions of a passive positioning alarm system.

## Methods

### Study design and sample

The present descriptive study was based on repeated informal conversational interviews [31] with eleven PwMDs living in their own homes. The participants were recruited with help from staff at one memory unit and one caregiver support centre for relatives in central Sweden. Inclusion criteria were having mild dementia, a need and desire to be alone outdoors and being able to participate in a conversational interview. The first and second interviews were conducted by the first author from June 2009 to April 2010.

### Data collection

The tape-recorded interviews were held in the participants’ homes; on the first occasion, the interviews lasted between 35 and 87 min (mean 56 min) and on the second occasion between 36 and 94 min (mean 52 min). A guide with open-ended questions was used [32] covering two topics: 1) PwMDs’ reflections on being outdoors (presented in [33]) and 2) their opinions on a PPA demonstrated during both interviews. The participants were during the interviews able to hold and test the device. Following the demonstration, the conversations continued with questions concerning, e.g., PwMDs’ reflections on the PPA and on integrity and self-determination when using it. Repeated interviews were used to enable participants to reflect on the PPA and their views on it; after seeing the PPA they may have had opportunities to observe similar aids in their surroundings, or on the news, etc. The repeated interviews were also considered valuable in establishing a trustful interviewer-interviewee relationship.

### System description

The PPA used is based on a global positioning system (GPS), including a transmitter (worn by the PwMD while alone outdoors) and a receiver (carried by the spouse, at home or elsewhere). The spouse activated the transmitter before the PwMD left the home (or other place), thereby creating a virtual fence with a radius of 500 m (a predefined area that could be changed and individually adjusted). As long as the PwMD stayed inside the predefined area, no alarm was sent. The minute the PwMD left the area, an alarm (a Short Message Service (SMS) containing a map with red dots representing the position of the transmitter) was sent to the spouse’s cell phone. The position of the transmitter was monitored every minute and sent to the receiver. If the PwMD left the predefined area and then returned to it, the red dots remained visible on the screen, even inside the virtual fence. In case the spouse needed to find the PwMD outdoors, an arrow was seen on the cell phone screen, pointing out the direction of the transmitter. However, there can still be problems in finding PwMD if the person moves quickly, due to that the device not constantly sends it position. One challenge with tracking technologies is to keep the device and especially the battery as small as possible to be feasible to use and carry with you on the other hand a small battery limits the possibility to constant send the position. For the present device communication was also possible, as the transmitter has a loudspeaker function and the spouse’s phone number can be programmed in. The PwMD could thereby, with one push of a button, get into contact with the spouse. As a further built-in safety feature, an SMS was sent to the receiver if the transmitter battery was low.

### Data analysis

All interviews were transcribed verbatim and analysed using qualitative content analysis [31]. Meaning units related to the study aim were identified, condensed, coded and deductively assigned to pre-determined categories based on Roger’s DOI Theory [29] and his description of attributes that are assumed to affect the diffusion and rate of adoption of innovations. To establish the study’s trustworthiness [31], the authors compared and had a critical discussion on each step in the analysis process. Credibility was also achieved by choosing participants of varying ages and both men and women, and quotations from the interviews are presented in the findings. The transferability of the findings to other contexts must be judged by the reader, based on his/her own experience, and through further research.

### Ethical considerations

The Regional Ethical Review Board at Uppsala University approved the study (reg. no. 2009/078). All participants received written and oral information about the study, and informed consent was obtained. Participation was strictly voluntary, and all participants were informed that they could withdraw from the study at any time without having to give an explanation.

## Results

Eleven PwMDs (mean age 68 years, range 62–72 years; 5 women, 6 men) participated in the repeated interviews; all had Alzheimer’s with a mean time since diagnosis of six years. Nine of the participants lived with a relative and two lived alone, although with relatives living nearby. All participants lived in a residential neighbourhood in a small town. Their average Mini Mental State Examination (MMSE) score was 25 (SD 3), and all participants except two (who used a cane and walker, respectively) managed their mobility without help. Housing environment varied across participants, with four PwMDs living in terrace houses, three in detached houses and four in apartments. Five of the participants also had holiday cottages. Overall, participants reported mixed perceptions of the PPA. The results from the deductive data analysis are organized according to Roger’s DOI Theory [29].

### Relative advantage

The participants described the value of the PPA in terms of increasing their and their relatives’ perceived feeling of safety and security: *“I feel safe, nice to know that he [relative] has an eye on me if I step outside it [the virtual fence] because then maybe I’m a bit lost… and if I’m in a fix, then it would just feel secure to know he knows where I am and if I missed a road”.* The informants also valued having the PPA for reasons other than being found. The PPA could be of assistance if one was outside and fell down or was injured in some other way and needed to contact relatives. The participants also mentioned the value of the PPA in their terms of increasing their own freedom and independence and at the same time the participants discussed the time aspect. One participant reported seeing no need for the PPA now, but reflected that it might be valuable in the future: *“Not because I think I need it now, but in the future it would probably feel good, having it is a form of security”*. The participants did not report fear of being supervised when carrying the alarm, but one participant reflected upon the PPA as a feeling of ‘big brother is watching you’. One participant reflected on use of the PPA as something that might enrich his quality of life: *“yeah… I guess it’s just as well, maybe I’ll get to live [a meaningful life] a little longer”.*

### Compatibility

The participants said that if and when they use the PPA should be their decision, and that introduction should start before their problems start, thus allowing them to decide for themselves: *“learning new things is hard… there will be more and more people like me [PwMDs] who have a diagnosis but still feel they’re pretty alert and who can still manage a lot, and then these aides [PPA] are great to have because then you can start using them before you get to ill, then you’ve already made that choice”.* The participants expressed concern about the cost of the PPA and who should pay for it. Some of the female participants also mentioned that use of the PPA could be seen as a “generational thing”; one women said: *“Just in general they [men] are more technology freaks than we women are, especially in this generation, but for the next generation it will be different because both sexes have grown up with technology. Then I thought, yeah, typically male, a little ‘calm security’”.* One female participant thought it might be easier to get men interested in starting to use the PPA: *“If they [men] get something technical and it’s explained as something technical, then that changes things, then there’s status involved or … I think it could work”.* Another female participant said: *“I mean men like machines so it shouldn’t be a problem, I don’t think, giving them one, getting them to think they’ve become technical, then it will work”.* Furthermore, the participants reflected on who might be the receiver of a potential alarm from the PPA. Some of them said that the only conceivable *“alarm recipient”* would be a relative (husband, wife or children), while for others this aspect was not important.

### Complexity

The participants reflected on how difficult the PPA might be to understand and/or use. Their thoughts alternated between possible problems with their own ability to manage the PPA and their relative’s potential difficulties. They also felt that the PPA should be easy to use and be perceived as easy to use: *“it [the PPA] is easier because a relative can just hang it on you if you go out, have it in your pocket…if you go outside the area then.. well, the alarms will sound anyway…”;* another participant said: *“that’s what the red cross is for, you can hardly miss it, and it’s known all over the world… really, I mean, red and it so noticeable that must be what you’re supposed to push”.* The participants also described the “*human factor”,* how people can be affected by technology, both positively and negatively, for example if one forgets to charge the cell phone or the transmitter, or if one of them is turned off: “*And then you have to have it [the PPA] on you too, not lying in a drawer somewhere”.* The participants also reflected on the fact that the PPA *“takes care of itself”* automatically: “*I don’t need to push anything when I go to daytime care centre, I just push the button if something happens, so we don’t have to be pushing a lot of buttons that makes it easier”*. The time aspect was considered important, that is, determining when the “right” time was to start using the PPA. As one participant stated: “*It may be best to start using the technology from the outset, jump on the bandwagon, so you don’t get left behind, you know, do it while your head is still clear”* and another said: “*Well, before you actually need it, so you’re familiar with it, know how it works and then comes the security part because this disease can progress pretty fast for some people”.* The participant said that learning to use and becoming familiar with the PPA should happen early in the disease trajectory, because it may be difficult during later stages of the disease.

### Triability

According to the participants, one prerequisite for future use of the PPA was having the opportunity to test the alarm while they believed they could still learn to use and handle it. They also said they needed to become ‘aware’ of the PPA and understand how it works. During the first and second interview, all participants held and tested (by pushing the buttons on the transmitter and receiver and, thus, generating a virtual fence) the PPA. Based on this ‘action’, the participants reflected on the PPA’s design and suggested things that needed to be changed, improved and complemented: “*can’t they add something so that when it [the PPA] hasn’t moved for a long time there’s a button, if you’re fully conscious you can active that button…”.* Furthermore*,”… if it had been a big device but it’s like a little ‘snuffbox’…”.* They reflected on the PPA’s stability/reliability, but also expressed some doubts in this regard: *“the more satellites he [the PPA] takes in, the closer you come to the right position, but I’m [the PwD] not 100 % sure anyway…*”. The participants sometimes made such observations spontaneously, but also upon reflection and in comparison with other technological support devices they were familiar with.

### Observability

The participants related the PPA to similar technologies they were familiar with. They said they had heard from friends and read in the press about similar technologies, “*the hunters I’ve heard about using it, they’ve been very pleased. I’ve seen guys [hunters] who have one [a PPA], there are many different kinds…”.* The participants reported having discussed future use of the PPA with family and friends. They had also described the PPA to other PwMDs at the daytime care centre and when visiting with friends and relatives: *“it [the PPA] is really good you know, because Lord how many people I’ve heard about over the years who’ve gone out for a walk and got lost and old people who disappear…”.*

## Discussion

The persons with mild dementia who were interviewed in the present study acknowledged that the PPA could be a useful tool. They had all experienced how the dementia symptoms had affected daily life. This may have influenced their attitudes towards and understanding of the potential value and usefulness of a PPA. Roger’s DOI Theory [29], which was used to analyse the interviews, revealed the importance of studying and grasping users’ experiences and then using these experiences when developing, implementing and managing the PPA from a person-centred perspective (cf. [34]). This means that innovative technical solutions, i.e., assistive technology such as a PPA, need to be developed and implemented *together with* potential users and not only *for* them. One important aspect revealed in our study concerned when and how the PPA should be introduced to PwMDs (cf. [35]). The participants could see the potential advantages of the PPA in terms of security and safety, although they felt it would provide suitable support in the future, when their daily life might be even more affected by dementia and difficult to handle (cf. [36, 37]). Earlier studies have also shown the importance of early introduction, thus allowing the PwMDs and their families to test and adapt to the technology while they still are capable of doing so (e.g., [11]). The participants valued the PPA, as it could give them security and independence and allow them to maintain activities and interests that still were important to them. Earlier interview studies [33, 38, 39] with PwMDs living in their own homes have revealed that being outdoors and being connected with the community were seen as vital to emotional well-being, interacting with other people and maintaining quality of life. In these contexts, use of a PPA would seem to be a way to promote the health and well-being of PwMDs (cf. [40–42]). The participants felt that the PPA gave them the ability to make immediate contact with their partner, which was seen as an important aspect of feeling safe. In our study, it proved useful to describe PPA-based safety facilitation using the components of Roger’s DOI Theory. *Compatibility* and *complexity* seemed to be part of a dual process were learning, testing (*trialability*) and handling the complexity of the PPA were followed by potential adoption of the PPA and, thereby, a feeling of control and safety. Some of the female participants in our study felt that using the PPA might be easier for men. This may be related to gender or more likely related to the older generation of women. The *compatibility* of the PPA seemed to be facilitated by individual adoption and followed up despite gender and generation. Costs are, however, a central aspect related to compatibility and to what degree a PPA can be made available to all (cf. [35]). *Observability*, the extent to which the innovation provides tangible results was as with other preventive innovations (cf. [43]), the attribute that were least mentioned.

The excitement surrounding technology-based interventions for PwMDs has not diminished, and research have moved to a new stage of identifying facilitators of and barriers to adoption of [11, 44, 45], implementation of [46] and adherence to technologies (cf. [46, 47]) to support daily life among PwMDs and their relatives. According to Roger [43] innovations that are perceived by individuals as having greater relative advantage, compatibility, trialability, observability, and less complexity will be adopted more rapidly than other innovations. Hence, the PPA has potential to be rapidly adopted and implemented under conditions that this technology is further adopted together with PwMD and their needs and experiences.

A limitation in the present study is that there are few participants and that they were not able to test the device between the interviews. Further studies are needed to better understand what security and privacy concerns users have and how these concerns can be addressed. Understanding the potential PPAs have for facilitating PwMDs’ outdoor activities, addressing accessibility issues inherent in tracking technology use, and educating providers about the benefits of PPA use are crucial to enhancing adoption and effective use of a PPA.

## Conclusions

Supporting PwMDs in their own homes using, e.g., a PPA enables them and their relatives to remain longer in their own homes and be safer in their own neighbourhoods. Future development of advanced technology for PwMDs should include potential users’ perceptions and experiences of the design prior to implementation. If this is done, we believe interventions will truly live up to their potential to support both PwMDs and their relatives.

## Availability of data and materials

Not applicable.

## Abbreviations

PwMDs: Persons with mild dementia
PPA: Passive positioning alarm
GPS: Global positioning system
SMS: Short message service
DOI: Diffusion of innovation
